# Statin Therapy as a Preventive Strategy for Nephrolithiasis: A Review of Current Evidence

**DOI:** 10.7759/cureus.93541

**Published:** 2025-09-30

**Authors:** Katie Gilman, Halley McDonald, David Addison, Daniela M Rizzo, John Ashurst, Pamela Potter, Carly Ernst-Kuhn

**Affiliations:** 1 Medicine, Midwestern University Arizona College of Osteopathic Medicine, Glendale, USA; 2 Medicine and Surgery, Midwestern University Arizona College of Osteopathic Medicine, Glendale, USA; 3 Research, Midwestern University Arizona College of Osteopathic Medicine, Glendale, USA; 4 Pharmacology, Midwestern University Arizona College of Osteopathic Medicine, Glendale, USA; 5 Clinical Education, Midwestern University Arizona College of Osteopathic Medicine, Glendale, USA

**Keywords:** anti-inflammatory, hmg- coa reductase inhibitors, kidney stone disease, oxidative stress, pleiotropic effects, prevention of kidney stones, recurrent kidney stones, recurrent nephrolithiasis, renal calculi, statins

## Abstract

Recurrent nephrolithiasis remains a significant clinical challenge due to the highly variable and multifactorial etiology of kidney stone formation, which complicates the development of effective preventive strategies. Statins, or 3-hydroxy-3-methylglutaryl coenzyme A (HMG-CoA) reductase inhibitors, are among the most widely prescribed medications, primarily for their cholesterol-lowering effects. Beyond their lipid-lowering properties, statins exhibit pleiotropic effects, including anti-inflammatory and antioxidative actions, that may contribute to reducing kidney stone formation. Moreover, statins are widely available, well-tolerated, and safe, highlighting them as a promising therapeutic option for nephrolithiasis prevention. Preclinical studies and observational data suggest that statins could play a role in preventing recurrent kidney stones. Despite encouraging preliminary findings, however, clinical evidence supporting the efficacy of statins in kidney stone prevention is currently limited. This review explores the potential mechanisms, preclinical findings, and current evidence regarding the use of statins as an adjunct therapy for recurrent nephrolithiasis and discusses the need for prospective clinical trials to investigate the efficacy of this therapy.

## Introduction and background

Nephrolithiasis, also termed kidney stones, is a condition characterized by the formation of solid crystalline masses within the urinary tract system, including the renal calyces and pelvis [1]. With a self-reported prevalence of 9.9% in U.S. adults and a recurrence rate of 50% within five years of the initial episode, nephrolithiasis is becoming increasingly more common [2,3]. Calcium-based stones (70%-80%) are most common, followed by uric acid (5%-10%), struvite (5%-15%), and cystine (<1%) stones [2]. Nephrolithiasis also imposes a substantial medical and economic burden, accounting for approximately $10 billion annually in healthcare costs associated with medication, inpatient and outpatient visits, and surgical interventions [4].  

The development of nephrolithiasis is multifactorial and involves a complex interplay of organic solutes, such as calcium, oxalate, and uric acid, that become supersaturated in urine [5]. The oversaturation of these organic materials promotes the nucleation and growth of crystals, which subsequently aggregate to form stones within the renal tubules and interstitium. The interactions between the endothelium and the crystals can induce epithelial cell injury, promoting the increased expression and synthesis of pro-inflammatory macromolecules and reactive oxygen species (ROS) [3]. This inflammatory process activates the NLRP3 inflammasome, releasing pro-inflammatory cytokines that further promote crystal aggregation and growth [6].  These processes generate crystal-induced oxidative stress that damages renal tubular cells, impairs endogenous anti-crystallization defenses, and accelerates stone formation.

The risk factors for kidney stone development involve a combination of genetic, metabolic, lifestyle, and dietary factors. Medical conditions such as type 2 diabetes, hypertension, obesity, and gout have been correlated with an increased risk of nephrolithiasis due to derangements in urinary pH, alterations in renal calcium excretion, and inflammation [7,8]. Due to the frequency, burden, and common risk factors associated with the disease, researchers have begun to look for medications that could be used as adjunct therapy to help diminish the recurrence or initial formation of nephrolithiasis. 

Statins inhibit HMG-CoA reductase, the rate-limiting enzyme in cholesterol biosynthesis, and are prescribed primarily for dyslipidemia [9]. By lowering low-density lipoprotein cholesterol (LDL-C), statins decrease atherosclerotic cardiovascular morbidity and mortality [10]. Beyond lipid reduction, statins inhibit small GTP-binding proteins (Rho, Ras, Rac), attenuating downstream inflammatory and oxidative pathways [8]. New research has shown that these commonly prescribed medications could not only improve metabolic profiles but also attenuate the oxidative stress and inflammation that lead to kidney stone development. The purpose of this review is to examine the potential mechanisms, preclinical findings, and existing evidence on the use of statins as an adjunct therapy for recurrent nephrolithiasis and identify where further investigation is necessary. 

## Review

Statins and their pleiotropic effects

While well-known for their cholesterol-lowering effects, statins also exhibit significant anti-inflammatory properties through various mechanisms. Several large-scale randomized controlled trials (RCTs) have demonstrated their ability to reduce C-reactive protein (CRP), a sensitive clinical marker of inflammation [11]. In vitro studies provide mechanistic insights, showing that simvastatin, atorvastatin, and lovastatin inhibit the DNA-binding activity of the pro-inflammatory transcription factor NF-κB [12,13]. Another pathway involves inhibition of L-mevalonate formation from HMG-CoA, which in turn limits the activity of Rac1, a G protein that mediates ROS production via nicotinamide adenine dinucleotide phosphate (NADPH) oxidase [14]. Additionally, atorvastatin has been found to downregulate angiotensin II type 1 receptor expression, leading to decreased superoxide release [15]. These molecular actions collectively demonstrate the therapeutic potential of statins, suggesting they may benefit conditions driven by inflammation and oxidative stress. 

Statins are also known to directly protect against endothelial dysfunction, thereby attenuating inflammatory processes. This protective effect is particularly important as endothelial dysfunction is a precursor to many cardiovascular and inflammatory diseases. Research has demonstrated that statins can upregulate endothelial nitric oxide synthase (eNOS) through multiple mechanisms, independent of their lipid-lowering properties [14,16]. By increasing eNOS activity, statins improve endothelial function and reduce vascular inflammation. Another mechanism through which statins exert their protective effects is by modulating cellular signaling pathways involved in the oxidative stress response. One such pathway involves nuclear factor erythroid 2-related factor 2 (Nrf2), a signaling molecule that upregulates the expression of antioxidant enzymes [17]. This function of Nrf2 is critical for counteracting oxidative damage in vascular tissues. Importantly, statins have been shown to enhance Nrf2 DNA-binding activity, further amplifying their antioxidative effects [18]. Collectively, these mechanisms illustrate how statins boost vascular health through complementary anti-inflammatory and antioxidative pathways.  

Statins may also offer protective effects against kidney stone disease, an area of growing interest in urologic research. The primary lipid-lowering effects of statins could play a role in this protection by improving lipid profiles associated with kidney stone risk. A retrospective study comparing the lipid profiles of stone-formers and controls found that elevated triglyceride levels and reduced HDL cholesterol were both significant risk factors for kidney stone disease [19]. These data suggest that correcting dyslipidemia may reduce the risk of stone formation. Supporting this, a recent cross-sectional analysis from the CDC’s National Health and Nutrition Examination Survey (NHANES) confirmed an increased risk of kidney stone disease associated with low HDL cholesterol levels [20]. Beyond these lipid-related effects, statins may also reduce kidney stone formation through unique mechanisms independent of their impact on cholesterol levels. One such mechanism involves mitigating lipid peroxidation, which has been linked to membrane damage via oxidative stress and urolithiasis in calcium oxalate (CaOx) stone disease [21]. Short-term atorvastatin therapy has been shown to lower plasma levels of malondialdehyde, another marker of oxidative stress  [22]. This effect likely reflects both reduced oxidized LDL and LDL-independent antioxidant actions. These findings highlight the potential of statins to address kidney stone disease through both lipid modulation and oxidative stress reduction. 

Since CaOx stones are the most prevalent type of kidney stones, their prevention should be a primary focus in managing kidney stone disease. For most patients, preventing CaOx crystal deposition is essential to reducing the risk of stone recurrence. Research using a rat model demonstrated that atorvastatin treatment significantly decreased the formation of renal crystal deposits [23]. This finding suggests that statins may have a direct protective effect on the kidneys in the context of stone disease. One proposed mechanism for this effect is the inhibition of the NADPH oxidase subunit NOX-1, which plays a key role in oxidative stress and renal tubular damage. By targeting NOX-1, statin therapy helps prevent tubular injury and reduces CaOx stone formation [24]. Additionally, atorvastatin has been shown to inhibit renal inflammation triggered by CaOx crystal deposition, further supporting its protective role in kidney stone disease [25]. This anti-inflammatory effect highlights the potential of statins to address multiple pathways involved in stone pathogenesis. Together, these findings suggest that statins could be a valuable adjunct therapy for preventing kidney stone recurrence.

Evidence for statin therapy in nephrolithiasis prevention

Multiple population-based studies have examined the association between statin use and the incidence of nephrolithiasis, revealing promising protective effects that suggest statin use may be linked to a reduced risk of kidney stone formation. In a large retrospective study, Sur et al. found that hyperlipidemic patients taking statins had significantly lower rates of nephrolithiasis compared to those not taking statins [26]. Further supporting this, another clinical retrospective study from Cohen et al. demonstrated that statins not only reduced stone formation overall but were particularly effective in recurrent stone-formers [27, 28]. Additionally, a meta-analysis of several observational studies linking dyslipidemia and urolithiasis found that patients with higher triglyceride levels and lower high-density lipoprotein (HDL) levels had an increased risk of kidney stone formation [29]. These findings underscore the interplay between lipid metabolism and kidney stone disease, reinforcing the potential utility of statins in reducing nephrolithiasis risk. 

However, limited experimental clinical data support the use of statins in kidney stone prevention. One prospective study found that atorvastatin use significantly reduced urine citrate levels in patients with high (≥20%) 10-year ASCVD risk [30]. Since urinary citrate is a known inhibitor of stone formation, these results are promising for the use of atorvastatin in stone prevention [31,32]. Another preliminary RCT from Taheri et al. investigated the effect of atorvastatin on markers of CaOx stone formation but did not find any significant effects [33]. This study used 24-hour urine and oxidative stress markers as primary outcomes rather than directly measuring the incidence of stone formation [33]. While this seems to contradict previous observational studies, the small sample size of just 28 participants in this RCT limits its ability to provide robust evidence for or against the use of statins in kidney stone prevention. Due to the lack of large-scale prospective studies, it remains difficult to determine whether statin use is causally related to reduced kidney stone recurrence or if the association is merely correlative. Although statins were not directly shown to reduce the risk of urinary stones, a Mendelian randomization study by Tan et al. did establish a causal relationship between elevated triglyceride levels and increased risk of urolithiasis [34]. Furthermore, multiple meta-analyses have confirmed that statins effectively lower triglyceride levels [35,36]. Therefore, it is plausible that the reduction in triglycerides by statins could be one causal mechanism through which they reduce the recurrence of nephrolithiasis. 

Randall’s plaques are deposits of calcium phosphate (CaP) that form in the renal papilla and serve as a nidus for CaOx kidney stone formation [37]. They are a critical focus of urology research, as they represent the initial site of idiopathic CaOx stone formation. These plaques develop due to urinary supersaturation in the renal tubules, which induces stress on the renal tubular epithelium, triggering inflammation and dysregulation of signaling pathways and subsequent plaque formation [37]. While current medical management primarily targets supersaturation, focusing on the inflammatory steps of stone pathogenesis could provide an alternative target for preventive therapies [38]. Supporting this approach, studies in animal models have demonstrated that reducing ROS formation significantly decreases crystal deposition [39]. This finding highlights the role of oxidative stress in stone formation and suggests that therapies targeting ROS could be effective. Statins, known to inhibit ROS formation [21,22], may directly intervene in the inflammatory processes underlying Randall’s plaque development, potentially halting one of the earliest steps in CaOx stone formation. Additionally, the vascular theory of Randall’s plaque formation posits that vascular injury could be the initiating event [40]. If this theory is correct, statins could provide further protection by mitigating vascular injury, offering a dual mechanism of action in preventing plaque formation. These findings collectively suggest that statins could play a role in preventing kidney stone formation by targeting both inflammatory and vascular pathways. 

Statins have demonstrated potential to inhibit calcium crystal deposition and reduce kidney stone formation in animal models. Liu et al. investigated the effects of atorvastatin on CaOx urolithiasis in hyperoxaluric rats with hyperlipidemia induced by a high-fat diet [41]. This study revealed that atorvastatin reduced renal CaOx deposits by restoring osteopontin expression, decreasing oxidative stress, and shifting urinary calcium crystal composition from CaOx to CaP [41]. These results build on earlier research in a rat model, which similarly showed that atorvastatin decreased renal crystal deposits [23]. Together, these studies suggest that statins may target multiple pathways involved in stone formation, including oxidative stress and crystal composition, offering a multifaceted approach to prevention. However, while these preclinical findings are promising, further research is urgently needed to explore the clinical relevance of these mechanisms in human populations. Large-scale trials are essential to determine whether statins can be effectively repurposed as adjunct therapies for kidney stone prevention, especially in patients with concurrent hyperlipidemia and recurrent nephrolithiasis.

Limitations and controversies

The evidence regarding the use of statins to prevent nephrolithiasis remains inconsistent due to variability in study designs, patient populations, and measured outcomes. Some studies suggest a protective effect of statins, such as the work by Sur et al., which found a reduced risk of stone formation in hyperlipidemic patients within a military healthcare system, indicating potential benefits in this specific population [25]. Similarly, Cohen et al. reported a protective effect but proposed that the mechanism was unrelated to the lipid-lowering properties of statins, highlighting the complexity of their role [27]. However, significant variability exists in the populations studied, limiting the generalizability of findings. For example, the Sur et al. study was conducted in a military healthcare system serving a distinct population, including active-duty members and retirees in the Southwestern United States, which may not represent the broader public. Additionally, Liu and Huang demonstrated that statins could alter urinary biochemistry by increasing urinary citrate and decreasing urinary uric acid, changes that theoretically reduce stone risk [30]. Despite these findings, the biochemical changes observed have not yet been conclusively linked to clinical outcomes, underscoring the need for further research. The conflicting results and limited generalizability highlight the need for well-designed, large-scale studies to better understand the role of statins in nephrolithiasis prevention. 

Despite the growing body of research on the potential role of statins in preventing nephrolithiasis, there remains a lack of large-scale RCTs specifically addressing this question. Most existing studies are observational and vary significantly in terms of design, patient populations, and outcomes, leading to inconsistent findings. Large-scale RCTs would provide the controlled environment needed to isolate the effects of statins from these confounding variables and assess their true efficacy in reducing kidney stone risk. Furthermore, such trials could explore dose-response relationships, identify subpopulations most likely to benefit, and evaluate long-term outcomes. Without robust RCT evidence, the role of statins in nephrolithiasis prevention remains speculative, underscoring the need for well-designed studies to resolve these uncertainties. Given the safety profile of statins, such trials are likely feasible from a risk-benefit standpoint.. The US Preventive Services Task Force found that there was little to no risk of adverse effects from statins across a variety of studies and conditions [42]. However, numerous potential drug-drug interactions - including those with ketoconazole, gemfibrozil, and grapefruit juice - must be considered when designing future trials. These interactions will need to be carefully considered when selecting and screening patient populations for further study [43].

Clinical implications and future directions

Statin therapy may hold significant potential as a preventive strategy for patients at high risk of developing nephrolithiasis. High-risk populations include individuals with recurrent nephrolithiasis, metabolic syndrome, or chronic inflammatory conditions [2,6]. As discussed in this review, evidence suggests that patients newly prescribed statins are less likely to develop nephrolithiasis compared to those not on statins, with the protective effect being particularly pronounced in individuals with a history of nephrolithiasis [27]. This finding underscores statins’ potential utility in managing recurrent stone disease, a notably challenging clinical problem. Statin use has been associated with a significantly reduced risk of nephrolithiasis in hyperlipidemic patients. A multivariate analysis reported an odds ratio (OR) of 0.51 after adjusting for confounding factors such as age, sex, and comorbidities, indicating a robust protective effect [25]. This finding is particularly relevant for patients with metabolic syndrome, who often present with hyperlipidemia and are predisposed to kidney stone formation. The protective mechanisms of statins may extend beyond lipid-lowering effects. Statins are known to attenuate cholesterol-induced inflammation in the kidney by inhibiting the activation of the NLRP3 inflammasome, a key mediator in chronic inflammatory pathways [44]. This anti-inflammatory property may be instrumental in reducing the risk of stone formation in patients with chronic inflammation, providing a dual benefit in managing both systemic and localized inflammatory processes. These findings highlight the potential for statins to serve as a targeted preventive strategy for nephrolithiasis in specific high-risk populations.  

Statins may enhance current preventive strategies for nephrolithiasis by offering a synergistic effect when combined with existing preventive measures. One potential mechanism is their ability to favorably alter urinary biochemistry, including increasing urinary citrate and pH while decreasing urinary uric acid levels, changes that are known to reduce the risk of stone formation [29]. This biochemical modulation could complement other preventative measures and provide an additional layer of protection against recurrent nephrolithiasis. Dietary therapy remains a cornerstone of nephrolithiasis prevention, with the American Urological Association recommending increased fluid intake to achieve a daily urine volume of at least 2.5 liters to dilute urinary solutes and reduce stone risk [45]. By lowering urinary calcium, statins may augment the protective impact of high fluid intake. This integration of pharmacologic and dietary interventions represents a holistic approach to managing nephrolithiasis. 

Although statin therapy shows promise as a preventive strategy for nephrolithiasis, further RCTs and mechanistic studies are essential to confirm its efficacy and safety. Current evidence is primarily derived from preclinical studies, with limited data from clinical trials to validate these findings in human populations. Establishing appropriate dosing regimens and determining patient populations most likely to benefit from statin therapy remain critical gaps in the literature. Notably, the AUA has not yet incorporated statin therapy into its guidelines for nephrolithiasis management, highlighting the need for more robust clinical evidence [45]. Future research should prioritize exploring the differential effects of various statins on nephrolithiasis prevention. To date, most preclinical studies have focused on atorvastatin, which has demonstrated efficacy in reducing free radical production and inhibiting renal inflammatory pathways associated with stone formation [22]. Investigating the potential of other statins, such as rosuvastatin or pravastatin, may reveal whether certain types offer superior protective effects or unique advantages in this context. Additionally, more research is needed to establish standardized dosing guidelines tailored to nephrolithiasis prevention. Understanding optimal dosage, duration, and patient-specific factors influencing efficacy will be crucial to translating these findings into clinical practice. Addressing these gaps through rigorous research will pave the way for integrating statin therapy into comprehensive guidelines for nephrolithiasis management. 

## Conclusions

The potential role of statins in preventing recurrent nephrolithiasis offers a promising avenue for improving the management of this challenging condition. Statins' pleiotropic anti-inflammatory and antioxidative properties, coupled with their wide availability and favorable safety profile, position statins as an attractive candidate for adjunctive therapy. Preclinical studies and observational data provide supportive evidence, suggesting that statins may reduce kidney stone formation by modulating biochemical pathways associated with stone pathogenesis. However, significant uncertainties remain, as current clinical evidence is limited and inconsistent, with studies often hindered by confounding factors and a lack of generalizability. While early findings are encouraging, the absence of large-scale RCTs leaves the true clinical efficacy of statins in nephrolithiasis prevention unproven. Addressing these gaps through rigorous research is essential to determine the mechanisms, optimal patient populations, and long-term benefits of statins in this context. Statins represent a potentially valuable addition to nephrolithiasis prevention strategies, but further investigation is needed to establish their utility in clinical practice. 
